# Hahnemann’s Birthday; the Banquet of the Lippe Society

**Published:** 1882-05

**Authors:** 


### HAHNEMANN'S BIRTHDAY.

### THE BANQUET OF THE LIPPE SOCIETY.

Monday, April 10th, was the 127th anniversary of the birth of Samuel Christian Friedrich Hahnemann, which took place April 10th, 1755, in Meissen, Saxony. The day was appropriately celebrated by the Lippe Society of this city, an organization composed of those new school physicians who adhere strictly to Homœopathy, by a meeting last evening and a banquet, at the residence of Dr. Adolph Lippe, whose name was given to the society in recognition of his staunch defense of the doctrines as handed down by the master. Among those who sat down to the banquet were: Dr. Adolph Lippe; Dr. Edward Bayard, of New York; Dr. M. Preston, of Norristown; Dr. C. Lippe, of New York; Prof. T. F. Allen, of New York; Dr. C. Carleton Smith; Dr. E. J. Lee; Dr. W. Jefferson Guernsey; Dr. M. MacFarlan; Dr. E. Payson Small; Dr. George H. Clark, of Philadelphia.

After partaking of the bountiful repast, an hour was very pleasantly spent in the presentation of appropriate toasts and in informal addresses. After the memory of Samuel Hahnemann had been proposed, Dr. Bayard responded to "The day we celebrate."

[Dr. Bayard's address is given in full at page 201.]

Dr. Clark, as Secretary of the Lippe Society, read a number of letters from distinguished homœopathists in different parts of the country, regretting their inability to be present, but assuring the society of their interest and sympathy. Dr. Wm. P. Wesselhœft, of Boston, wrote: "I regret exceedingly not to be able to attend the Hahnemann birthday celebration of the Lippe Society. I shall be with you in spirit and give you this sentiment:

"*Samuel Hahnemann*, the discoverer of the infinitesimal dose in disease, still derided by adversaries and ridiculed by so-called followers, must become the acknowledged pioneer of a new era in solving the problems of the physical laws of matter. *Adolph Lippe*, the worthy disciple, the most intrepid defender of the truths of his master's discoveries."

Dr. C. Pearson, of Washington, expressed the following sentiments in his letter: "While the hollow friends of Hahnemann are disposed to cast reproach on his fair fame and useful labors, it is refreshing to know that somewhere in the wide world there can be found at least twelve of his disciples meeting together without a Judas. Though the outlook may be dark and threatening, and the enemies of pure Homœopathy, with their pathological leaders at their head, swarm like Egyptian locusts, each armed with a microscope, however powerful, vastly incapable of discovering their own souls,—the inductive philosophy of Hahnemann will only shine the brighter when the mists of doubt and disparagement have passed away. It is only when the sea is rough that the beacon light is needed; let those who heed it not perish by their own folly if they will, but let it still burn for others. Stand by your guns, and by the flag of Hahnemann, and if we go down let it be with his banner still floating in the sunshine of truth."

In response to the toast, "The Pioneers of Homœopathy in America, dead and living; they who fought the good fight and have kept the faith," Dr. C. Carleton Smith said: "Though we come together to-night more especially to do homage to the name and genius of Hahnemann, yet we must not forget on this momentous occasion the pioneers of our school, who so faithfully prepared the way for the struggling disciples who followed closely in their footsteps. As we sit here with the spirit of Hahnemann hovering over us, our minds go back to the time when such valiant defenders of the faith as Hering, Gardiner and Esrey, of Philadelphia; Wesselhœft, of Boston; Joslin, Reissig, Hunt, Dunham, Gray and Kirby, of New York; Frietag, of Bethlehem; Bauer and Pulte, of Cincinnati; Temple, of St. Louis; Groseweitch, of Wilmington; Campos, of Norfolk, and Lingen, of Savannah, stood in the very heat of the battle, fighting nobly for the truth. Of the old pioneers still struggling and contending for the right, we have Drs. Lippe, Wells, Bayard, Pomeroy, Fellger, Guernsey, Kitchen, Raue, Detwiler and McManus. We would, brethren, be recreant to our duty to-night if we failed to ascribe glory and honor to these staunch defenders of the truth as it was handed down to us by the master.

"These men, both the dead and living, have stood as giant sentinels at the door of the temple, guarding well its sacred portals amid the gibes and sneers of the mongrels who despise the truth, and in their bitterness daily slander the good name of Samuel Christian Hahnemann, the latchets of whose shoes they are not worthy to unloose—and who would, if they had the power, bury out of sight beyond all hope of resurrection the Homœopathic bible, the *Organon*. What, I ask, would have been the condition of our school to-day, if these noble, self-sacrificing men, the advance guard of our army of healers, had not been staunch and true in every emergency, through good report and through evil report? Oh, let us write their names high upon the scroll of fame, and keep their memories green in our hearts. And though bigotry and superstition, ignorance and uncharitableness have uttered their words of detraction and have pointed their poisoned arrows at the very hearts of these heroes, and have sought to drive them from the right of conscience, yet the dead among them died unmoved and the living stand unmoved to-day—strong in the faith that the *right* must triumph, and that *similia* will ere long be acknowledged as *the only law of cure* by *all* the fraternity of medicine."

Dr. Fellger offered the following sentiment: "The Internationals: the guardians of the truth, who stand up fearlessly against the worst enemy of mankind—IGNORANCE."

Dr. Guernsey made a brief impromptu address in answer to the toast: "The International Hahnemann Association; the guardians of the truth," and Dr. Lee acknowledged "THE HOMŒOPATHIC PHYSICIAN: the promoter of the truth; may it long continue its good course."

The next toast was: "The Hahnemannian Tri-color; the Law of the Similars, the Single Remedy and the Minimum Dose." To this Dr. Lippe responded. He said:

"Hahnemann unfurled the tri-colored banner at the beginning of this century; on it were written 'the Law of the Similars, the Single Remedy and the Minimum Dose,' that he might conquer and annihilate the ever-changing opinions held out by the common school of medicine, all based on mere hypothesis, and replace all hypotheses, antiquated and modern, by such fundamental principles and by such laws of cure as were derived from the laws of nature, and proved to be correct by the experiment. He unfolded this banner for full recognition by the medical world, and his faithful followers will fight it out on that line, even if it takes another century."

Prof. Allen was called upon to respond to the toast: "The Materia Medica Pura: the Hahnemannian's armament, potent only for good." Dr. Allen said, that while others had spoken of curing disease by the principle of similia, Hahnemann was the first to elaborate a system of medicine on that principle, and to give the world a pure materia medica. The speaker then went on to mention how many elements of empiricism had entered the materia medica, and to enforce the necessity of thorough drug-proving as our only sure guide to the use of a drug as a curative. He said that while a clinical symptom needed frequent corroboration to make us sure of its worth, a pathogenetic symptom needed none; that we *knew* if a drug produced a symptom it would cure it. Hence the pathogenetic symptom has the advantage in being more reliable and more scientific. The professor then referred to the care and labor bestowed on his encyclopædia, and regretted that unavoidable errors should have crept in; yet he thought the vast majority of the symptoms there recorded were reliable. He also referred to the great care and labor bestowed by Hahnemann on his provings; how few errors had crept in, and said that nearly all of Hahnemann's clinical symptoms had been corroborated in more recent provings. Instancing the symptom—burning pain, etc., in the back, of Phosphorus, which had never been produced, though often cured, by Phosphorus, until Anstie, of England, gave the drug in large doses.

Dr. Allen referred to the practical work true homœopathists were doing, but cautioned his brethren against the many fragmentary and imperfect provings which are constantly being published.

The success of the Lippe Society was proposed by Prof. Allen, and responded to by Dr. Clark, and after several informal addresses the guests dispersed, very much pleased with the evening's entertainment and more determined than ever to hold fast to pure Homœopathy.—*Philadelphia Bulletin.*

